# Prolonging working life among blue-collar workers: The buffering effect of psychosocial job resources on the association between physically demanding and hazardous work and retirement timing

**DOI:** 10.1016/j.ssmph.2023.101372

**Published:** 2023-02-24

**Authors:** Johanna Stengård, Constanze Leineweber, Hui-Xin Wang

**Affiliations:** Stress Research Institute, Department of Psychology, Stockholm University, Stockholm, Sweden

**Keywords:** Retirement timing, Physically demanding work tasks, Physically hazardous work environment, Job resources, Moderator, Interaction

## Abstract

The need to delay retirement timing has been acknowledged in Western countries due to demographic ageing. The aim of the present study was to examine the buffering effects of job resources (decision authority, social support, work-time control, and rewards) on the association of exposures to physically demanding work tasks and physically hazardous work environment with non-disability retirement timing. Results from discrete-time event history analyses, in a sample of blue-collar workers (n = 1741; 2792 observations) from the nationwide longitudinal Swedish Longitudinal Occupational Survey of Health (SLOSH), supported that decision authority and social support may buffer the negative impact of heavy physical demands on working longer (continuing working vs retiring). Stratified analyses by gender showed that the buffering effect of decision authority remained statistically significant for men, while that of social support remained statistically significant for women. Moreover, an age effect was displayed, such that a buffering effect of social support on the association of heavy physical demands and high physical hazards with working longer were found among older men (≥64 years), but not younger (59–63 years). The findings suggest that heavy physical demands should be reduced, however, when not feasible physical demands should be accompanied by social support at work for delaying retirement.

## Highlights

• Social support enables blue-collar women with high physical demands to work longer.
• Social support enables blue-collar men aged ≥64 with high hazards to work longer.
• Social support enables blue-collar men ≥64 with high physical demands to work longer.
• Decision authority enables blue-collar men with high physical demands to work longer.

## 1 Introduction

Due to demographic ageing, governments in many countries aim to delay retirement timing. To understand work environment factors that can prolong a sustainable working life are therefore of great importance. Blue-collar workers are a particularly important group to study. Besides that they are at a substantial larger risk compared to white-collar workers for premature exit from the labour market due to disability pension or death (Kadefors et al., 2018), they do more seldom continue working after normative retirement ages (Leinonen et al., 2020; Virtanen et al., 2017). Also, blue-collar workers in general have both higher levels of physically demanding work tasks (physical demands) and more exposures to physically hazardous work environment (physical hazards). Previous research indicates that less physically demanding or hazardous work may matter for delaying old-age retirement timing (Andersen et al., 2021; Anxo et al., 2019; Böckerman & Ilmakunnas, 2020; Stengård, Virtanen, Leineweber, Westerlund, & Wang, 2022; Virtanen et al., 2017). Also, particularly high job resources are of significance in this regard (Browne et al., 2019), however to date, hardly any study has examined the possible buffering effects of job resources on the association between physically demanding or hazardous work and old-age retirement timing.

According to the Job Demands–Resources (JD–R) theory, job resources may: 1) stimulate personal growth and help achieve individual's goals, and 2) reduce physiological and psychological costs related to job demands (Bakker & Demerouti, 2007). Previous studies support the first assumption by showing an association of psychosocial job resources with later retirement (Andersen et al., 2021; Carr et al., 2016; Stengård et al., 2021; Van Solinge & Henkens, 2014; Virtanen et al., 2014). With regard to the second assumption, many studies support that job resources may buffer negative health effects of job demands (Amiri & Behnezhad, 2020a, 2020b; Duchaine et al., 2020). Therefore, it is reasonable to expect that psychosocial job resources – for example, good social support or high control over work – may attenuate the impact of high physical job demands on retirement timing, such that, despite high physical demands, workers would prolong their working lives if they have good job resources. For example, having control over when and how to perform one's job, feeling social support from manager and colleagues, or being adequately compensated for one's physical efforts, may ease the impact of high physical demands and hazards, and have implications on one's ability and one's willingness to continue working yet another year. However, whether psychosocial job resources, such as decision authority, social support, work-time control, and rewards, have buffering effects on the association between physical job demands and old-age retirement timing remain unknown. To our knowledge no study has examined the role of psychosocial job resources in the relation between physical hazards and old-age retirement timing and only one previous study has investigated interactive effects between psychosocial job resources and physical demands on working beyond 65 years (Andersen et al., 2021).

Comparing seated vs physically active work, job resources status did not influence the effect of physical demands on retirement timing (Andersen et al., 2021). However, only single-item questions were used to measure exposures up to ten years before retirement timing was measured. More importantly, as the majority of white-collar workers have no or only low physical demands, when including both blue- and white-collar workers, they basically compared blue-with white-collar workers.

In addition to higher job resources being associated with later old-age retirement, one Swedish study found that job resources, such as work-time control, skill use, and rewards, grow in importance with age for continuing work among individuals who had reached pensionable age (Stengård et al., 2021). Another study found that physical demands was more strongly associated with retirement for the oldest in a sample of men aged at least 62 years (McLaughlin & Neumark, 2018). If there in fact is a buffering effect of job resources on the influence of physically demanding work on retirement timing, it is reasonable that, with age, more job resources are needed to buffer heavy physical demands and high physical hazards.

**Fig. 1:**
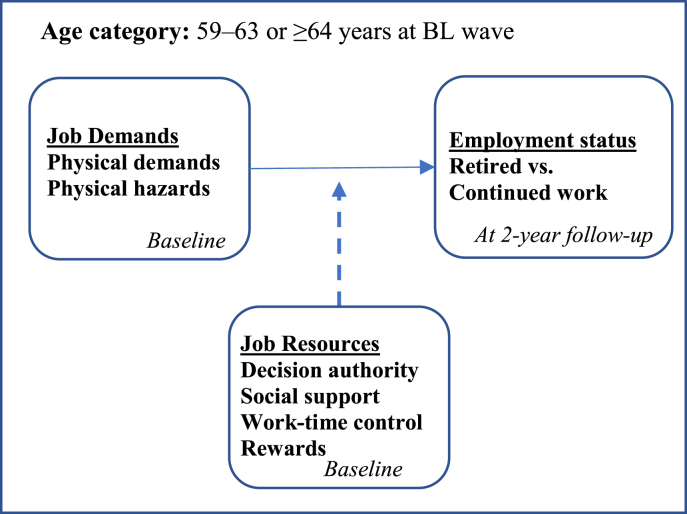
Conceptual model of the study's aims. Age categories 59–63 and ≥ 64 years, respectively. Stratified by gender.

The aims of the present study were to examine the role of job resources in the association of physical demands and physical hazards with working longer among blue-collar workers, and whether the role of job resources on the association was stronger for older male and female workers than younger workers, respectively. Because the Swedish labour market is rather gender-segregated (Cérdas et al., 2019), women and men to a large extent are working in different sectors and men tend to work longer (Myllyntausta et al., 2021). Stratifying analyses by gender would provide important information on the influence of the interplay between demands and resources on retirement timing for men and women, respectively. The conceptual model of the present study's aims is presented in Fig. 1.

## 2 Methods

### 2.1 Sample and procedure

The present study was based on the nationwide Swedish Longitudinal Occupational Survey of Health (SLOSH) cohort study, a follow-up study of the Swedish Work Environment Surveys (SWES, 2003–2011) conducted biennially by Statistics Sweden. The SWES consists of a subsample of gainfully employed people aged 16–64 from the Labour Force Survey (LFS). These individuals were first sampled into LFS through stratification by county, sex, citizenship and inferred employment status. Thus, SLOSH is approximately representative for the Swedish working population, which has been followed-up biennially since 2006. There are two versions of the SLOSH questionnaire: 1) for those who are currently working (at least 30% of a full-time job) or 2) for those who are not working at all or working less than 30% of a full-time job. In 2020, SLOSH comprised over 40,000 individuals. The SLOSH and the present study have been approved by the Regional Research Ethics Board in Stockholm. A detailed description of SLOSH is provided elsewhere (Magnusson Hanson et al., 2018).

The study population is derived from the SLOSH 2008–2018 surveys. Individuals were included in the study if they (i) responded to the questionnaire for those currently in work at least once between 2008 and 2016 and two years later had answered either the questionnaire for workers or for non-workers and stated being full-time retired with old-age pension, (ii) were at least 59 years when answering the questionnaire (thus they reached the lowest state pension age of 61 years at the follow-up wave), (iii) were blue-collar workers (unskilled and skilled manual workers), and (iv) were not self-employed. The effective sample did not differ from the dropouts (i.e., non-respondents at the subsequent follow-up) in terms of levels of physical job demands and job resources.

To investigate the influence of physical job demands and hazards and their interplay with job resources on retirement timing, participants were followed in 2-year intervals. These pair of waves (baseline/follow-up) build one observation on retirement status (e.g. work-work or work-retirement), where psychosocial working conditions and covariates were assessed at the baseline wave and the retirement status at the follow-up. An observation is defined by any baseline wave during 2008–2016 and the subsequent (2-years later) follow-up wave during 2010–2018. A person could contribute with up to five observations (pairs of waves) at maximum. In the case that a retired person got re-employed, only the first “from work to retirement” transition was considered. Waves where a person works less than 30% were not considered. The final analytical dataset comprised of 2792 observations from 1741 individuals who had completed a “from work to retirement” transition or at least one “work to work” (either remaining in the same job or transferring to another one) transition between two subsequent waves.

#### 2.1.1 Setting the scene

The Swedish context is suitable for examining interactive effects between physical demands or physical hazards and job resources on retirement timing, as the Swedish pension system has no fixed statutory retirement age, although, 65 years is a rather strong normative retirement age (Anxo et al., 2017; SOU, 2020, p. 69). In 2018, the actual average age for retirement in Sweden was for women 63.6 and for men 64.6 years (SOU, 2020, p. 69). Up to 2019, it was feasible to retire (part- or full-time) and start receiving collected earnings-related state and civil servant pension from the age of 61. Guarantee pension, which only is paid to individuals with low collected earnings, was paid from 65. Employees had legal rights to keep their employment until their 67th birthday, thereafter the individual could continue in agreements with the employer (https://www.pensionsmyndigheten.se).

### 2.2 Measures

#### 2.2.1 Outcome variable

*Working longer:* continued work (1) versus retired (0) within the next two years (measured at the follow-up wave). Individuals were regarded as still working if they responded to the questionnaire for workers (i.e., working at least 30% of a full-time) and retired if they responded to the questionnaire for non-workers and stated being full-time retired with old-age pension.

#### 2.2.2 Exposure variables

*Physical work characteristics* were assessed with two scales: physically demanding work tasks and physically hazardous work environment. Both scales had response alternatives 1) “not at all”, 2) “a little, perhaps 1/10 of the time”, 3) “about ¼ of the time”, 4) “about half of the time”, 5) “about ¾ of the time”, or, 6) “almost all the time”. *Physical demands* were assessed with a three-item scale (physical labour, heavy lifting, and awkward working positions) (Åkerstedt et al., 2015). Cronbach's alpha was 0.84. A sum-index was estimated with score range 3–18. Physical demands were dichotomized into a binary indicator according to the median value: 0 “light physical demands” (53.4%; score range 3–9) or 1 “heavy physical demands” (46.6%; value range 10–18). *Physical hazards* were assessed with six items that focused on whether the individual was exposed to any of the following at work: 1. noise, 2. poor or excessively bright light, 3. excessive heat, cold or draught, 4. vibrations that make your whole body shake and vibrate, 5. toxins or irritants, and, 6. tangible risk of injury (Swedish Work Environment Authority, 2016, p. 2). Cronbach's alpha was 0.78. First, each of the item scores was dichotomized: 0 not or little exposed (score 1–2) versus 1 exposed (score range 3–6). Thereafter, a sum-index was estimated with value range 0–6 indicators. Finally, the scale was dichotomized into a binary indicator according to the median value: 0 “low physical hazards” (53.8%; 0–1 indicators) or 1 “high physical hazards” (46.2%; 2–6 indicators).

#### 2.2.3 Moderators

*Job resources* were assessed with four scales: decision authority, social support, work-time control, and reward. Decision authority and social support were derived from the reliable and well-tested Demand–Control–Support-Questionnaire (DCSQ) (Chungkham et al., 2013; Theorell et al., 1988), with response alternative on a 4-degree Likert scale ranging from 1: No, (almost) never to 4: Yes, often. *Decision authority* was measured with a 2-item-scale (what to do at work and how to do the work). The Scale reliability coefficient was 0.76. *Social support* was assessed with a 5-item-scale regarding pleasant atmosphere, understanding and cohesiveness among co-workers and managers. Cronbach's alpha was 0.85. *Work-time control* was measured with a 6-item-scale (Ala-Mursula et al., 2002), assessing the opportunities to influence the working time (length of work day, start and end times, taking breaks, running private errands during worktime, which days to work, and vacations) with five response alternatives ranging from 1: no, to a very small extent to 5: yes, to a large extent. Cronbach's alpha was 0.82. *Rewards* were measured by a 7-item-scale (Li et al., 2019) concerning job promotions (adequate salary, work and promotion prospects), esteem (receiving the earned acknowledgement, prestige and respect), and job security (including not expecting or experiences undesirable job changes) with response alternative on a 4-degree Likert scale ranging from 1: no, not at all to 4: yes, completely agree. Cronbach's alpha was 0.64.

A sum-index was estimated for each job resource. Thereafter, they were dichotomized into binary indicators according to their respective median values: decision authority: 0 “low” (37.5%; score range 2–5) or 1 “high” (62.5%; score range 6–8), social support: 0 “low” (51.5%; score range 5–15) or 1 “high” (48.5%; score range 16–20), work-time control: 0 “low” (52.5%; score range 6–13) or 1 “high” (47.5%; score range 14–30), and rewards: 0 “low” (44.7%; score range 7–17) or 1 “high” (55.3%; score range 18–28) (Table 1). Job resources were used as moderator variables in the association between physical demands or physical hazards and working longer.

**Table 1:** Descriptive statistics and bivariate correlations with regard to the observations.

	1	2	3	4	5	N_obs_	N_lowest_ (%)	Cut-off value (range)
1 Physical demands	–					2729	53.39	10 (3–18)
2 Physical hazards	.279***	–				2696	53.75	2 (0–6)
3 Decision authority	−.035†	−.073***	–			2744	37.46	6 (2–8)
4 Social support	−.055**	−.147***	.144***	–		2669	51.48	16 (5–20)
5 WTC	−.028	.023	.244***	.100***	–	2593	52.45	14 (6–30)
6 Rewards	−.067**	−.101***	.181***	.306***	.157***	2262	44.69	18 (7–28)

*Note*. All variables are dichotomized. N_obs_ for number of observations (transitions). N_lowest_ for the percent of observations in the category below the cut-off value. *** for *p* < .001; ** for *p* < .01; * for *p* < .05; † for 0.05 ≤ *p* < .10.

*Age category* (59–63 years (0) versus ≥64 years (1) at baseline). This cut-off was based on the fact that the older age category included observations where the individual approached 65 years (representing the Swedish normative retirement age) and consequently two years later (at follow-up) had passed the normative retirement age.

#### 2.2.4 Covariates

Age has a strong relation to retirement timing (Fisher et al., 2016) and, thus, in all the analyses age (in years) served as the timing of the event. Sociodemographic variables, such as gender, education level and family situation, have been shown to relate to retirement timing (Fisher et al., 2016) and hence we included gender, education level (four levels), marital status (married/cohabitant vs single), parental status (having children living at home vs not) and caring for a relative (yes vs no), as covariates (Table 2). We also controlled for part-time job (full vs part-time) which may attenuate heavy physical demands and influence retirement timing (Thorsen et al., 2016). All covariates were assessed at the baseline wave of each particular observation. Also, year of the questionnaire (wave; categorical) was utilized as a covariate, allowing for adjustment for potential timing effects. All the covariates were based on questionnaire data, except for age, gender, and education level which were fetched from registry data.

**Table 2:** Distribution covariates.

Variable	All observations	Women observations	Men observations
	2551	1209	1342
Gender			
Women (0)	1209 (47.4%)	n/a	n/a
Men (1)	1342 (52.6%)	n/a	n/a
Marital status			
Single (0)	613 (24.0%)	334 (27.6%)	279 (20.8%)
Married/cohabitant (1)	1938 (76.0%)	875 (72.4%)	1063 (79.2%)
Children living at home			
No (0)	2331 (91.4%)	1133 (93.7%)	1198 (89.3%)
Yes (1)	220 (8.6%)	76 (6.3%)	144 (10.7%)
Caring for a relative			
No (0)	2196 (86.1%)	999 (82.6%)	1197 (89.2%)
Yes (1)	355 (13.9%)	210 (17.4%)	145 (10.8%)
Part-time job			
Part-time (0)	844 (33.1%)	643 (53.2%)	201 (15.0%)
Full-time (1)	1707 (66.9%)	566 (46.8%)	1141 (85.0%)
Education level			
Elementary school (1)	679 (26.6%)	226 (18.7%)	453 (33.8%)
Secondary school (2)	1655 (64.9%)	868 (71.79%)	787 (58.6%)
Short higher education (3)	80 (3.1%)	29 (2.4%)	51 (3.8%)
University (4)	137 (5.4%)	86 (7.1%)	51 (3.8%)
Wave			
1st	374 (14.7%)	169 (14.0%)	205 (15.3%)
2nd	354 (13.9%)	158 (13.1%)	196 (14.6%)
3rd	347 (13.6%)	160 (13.2%)	187 (13.9%)
4th	764 (30.0%)	359 (29.7%)	405 (30.2%)
5th	712 (27.9%)	363 (30.0%)	349 (26.0%)

### 2.3 Statistical analysis

Discrete-time event history analyses with the binary outcome variable working longer (continued work vs retired) measured at follow-up were performed in Stata version 17.0 (xtlogit command). Age served as the timing of the event and was entered as a categorical variable in all models, inclusive the crude models (Model 1). For each person, the data admitted one to five transitions (observations; from-work-to-work or from-work-to-retirement) between two successive waves (baseline and the follow-up wave). The adjusted models (Model 2–4) were further adjusted for wave, gender, educational level, part-time job, marital status, parental status, and caring for a relative (all measured at the baseline wave of a pair of waves).

#### 2.3.1 Interaction effects

The moderating effect of a job resource on the association between physical demands/physical hazards and retirement timing was estimated by including a two-way interaction term (Models 1 and 2). All analyses were stratified by gender. To study whether the interaction between physical demands/physical hazards and job resources on retirement timing differed by age, a three-way interaction term between age category (59–63 versus ≥64 at baseline), job resource, and physical demands or physical hazards were included (Model 3). A statistically significant three-way interaction term (tested by a Wald χ^2^−test) indicates that the two age groups differ with regard to the moderating effect of the particular job resource on the association between physical demands/physical hazards and retirement timing. Thus, it is meaningful to further stratify the analysis by age category (Model 4).

## 3 Results

Among 1741 individuals who participated in their respective first survey (time point varies between the participants), 49% were women, 51% men, 67% worked full-time and 33% part-time (at least 30% of a full-time). Moreover, 77% were married/cohabiting, 9% had children living at home and 14% were caring for a relative. 27 percent had elementary school education, 66% secondary school education, 3% short higher education, and 5% university education. The mean age at the initial survey was 60.9 (SD 2.0) years ranging from 59 to 70 years. Descriptive statistics for the study variables (observations) can be find in Table 1, Table 2, Table 3.

**Table 3:** Distribution of the observations according to age category (59–63 or ≥ 64 years) and working longer status (continued work vs retired).

Age at baseline	Continued work at follow-up N (N_women_; N_men_)	Retired at follow-up N (N_women_; N_men_)	Total N (N_women_; N_men_)
59–63	1731 (852; 879)	507 (266; 241)	2238 (1118; 1120)
≥64	162 (54; 108)	392 (174; 218)	554 (228; 326)
Total	1893 (906; 987)	899 (440; 459)	2792 (1346; 1446)

*Note*. There were very few observations of women in the age category ≥64 who reported continued work at the subsequent follow-up wave two years later.

### 3.1 Association between physical demands/physical hazards and working longer

Initial tests on the main effects of physical demands and physical hazards in separate models showed that both light (vs heavy) physical demands and low (vs high) physical hazards were significantly associated with increased likelihood of working longer (Table 4) in the adjusted models. Among job resources, high (vs low) social support significantly increased the likelihood of working longer, while the contribution of high (vs low) work-time control was borderline significant (*p* = .093) in the adjusted model. Decision authority and rewards did not associate with working longer.

**Table 4:** Main effects of the study variables in separate models. Odds ratio (OR) and 95% confidence interval (CI) of working longer (continued work (1) vs retired (0) two years later).

	Crude model^a^	N_obs_ (N_ind_)	Adjusted^b^	N_obs_ (N_ind_)
Physical demands	0.79 (.64–.96)*	2723 (1717)	0.79 (.64–.98)*	2518 (1632)
Physical hazards	0.93 (.76–1.14)	2689 (1699)	**0.80 (.63**–1.00)*	2493 (1614)
Decision authority	0.97 (.79–1.20)	2738 (1724)	0.98 (.79–1.23)	2531 (1640)
Social support	**1.29 (1.05**–1.59)*	2666 (1691)	**1.37 (1.10**–1.70)**	2476 (1607)
Work-time control	**1.27 (1.03**–1.56)*	2589 (1656)	1.21 (.97–1.52)†	2407 (1574)
Reward	1.07 (.86–1.34)	2256 (1495)	1.11 (.88–1.41)	2099 (1419)

Note.

N_obs_ = number of observations; N_ind_ = number of individuals.

* for *p* < .05; † for 0.05 ≤ *p* < .10.

a Crude (age served as the timing of the event).

b Adjusted for age, wave, gender, education, marital status, parental status, part-time job, and caring for relative.

### 3.2 Effect of job resources on the association between physical demands and working longer

**Fig. 2:**
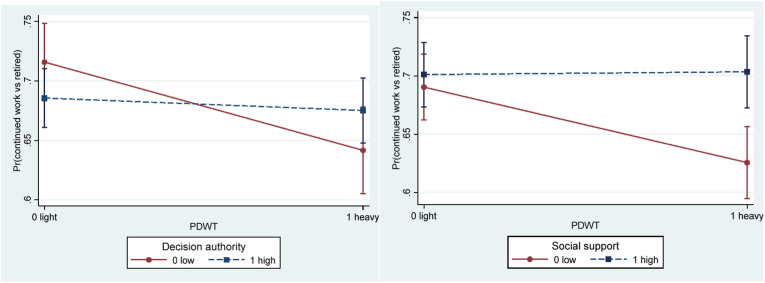
Predictive margins of continued work (vs retired) at follow-up, with 95% confidence intervals of interactive effects between physical demands and **a**) decision authority, and **b**) social support, in adjusted models. *Note.* PDWT for physically demanding work tasks (physical demands).

**Fig. 3:**
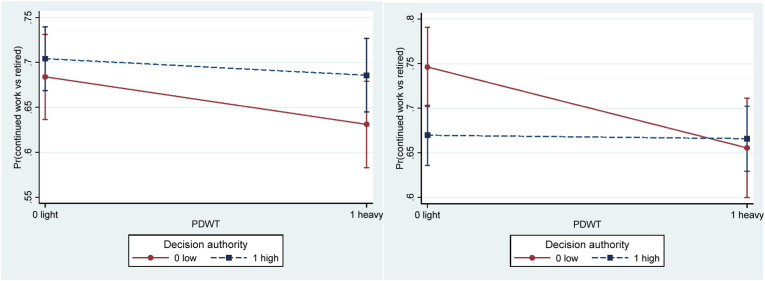
Predictive margins of continued work (vs retired) at follow-up with 95% confidence intervals of interactive effects between physical demands and decision authority for **a**) women (*p* = 0.454) and **b**) men *(p* = 0.048). *Note.* PDWT for physically demanding work tasks (physical demands).

Decision authority did not moderate the association between physical demands and working longer in the crude model (Table 5), but in the adjusted model the interaction term reached statistical significance (OR = 1.59, 95%CI 1.02–2.50). This result suggests that decision authority may buffer the negative influence of heavy physical demands on the probability of working longer. More specifically, among those with low (but not among those with high) decision authority there was a negative influence of heavy compared to light physical demands on the probability of working longer (Fig. 2a). After stratifying by gender, similar results were found among men (OR = 1.91, 95%CI 1.00–3.63), but not among women (OR = 1.28, 95%CI 0.67–2.46) (Table 6; Fig. 3).

**Table 5:** Interactive effect of job resources and physical demands in separate models. Odds ratio (OR) and 95% confidence interval (CI) of working longer (continued work (1) vs retired (0) two years later).

	Model 1.Crude	N_obs_ (N_ind_)	Model 2.Adjusted	N_obs_ (N_ind_)
Decision authority		2688 (1704)		2487 (1620)
DA	0.85 (.63–1.13)		0.80 (.59–1.09)	
PDWT	0.65 (.46–.91)*		0.58 (.41–.83)**	
DA*PDWT	1.36 (.89–2.07)		**1.59 (1.02**–2.50)*	

Social support		2616 (1671)		2432 (1587)
SS	1.09 (.82–1.44)		1.08 (.80–1.46)	
PDWT	0.67 (.50–.89)**		0.62 (.46–.85)**	
SS*PDWT	**1.45 (.95**–**2.19)†**		**1.63 (1.05**–2.53)*	

Work-time control		2542 (1638)		2365 (1555)
WTC	1.30 (.98–1.73)**†**		1.19 (.88–1.61)	
PDWT	0.81 (.61–1.09)		0.79 (.58–1.07)	
WTC*PDWT	0.95 (.62–1.44)		1.02 (.66–1.59)	

Reward		2217 (1479)		2065 (1402)
Reward	0.99 (.72–1.35)		1.02 (.74–1.41)	
PDWT	0.66 (.47–.93)*		0.66 (.46–.94)*	
Reward*PDWT	1.14 (.73–1.80)		1.18 (.74–1.91)	

Note.

Model 1. Crude (age served as the timing of the event).

Model 2. Adjusted for age, wave, gender, education, marital status, parental status, part-time job, and caring for relative.

DA for decision authority; PDWT for physically demanding work tasks (physical demands).

N_obs_ = number of observations; N_ind_ = number of individuals.

* for *p* < .05; † for 0.05 ≤ *p* < .10.

**Table 6:** Interactive effect of job resources and physical demands in separate models **stratified on gender.** Odds ratio (OR) and 95% confidence interval (CI) of working longer (continued work (1) vs retired (0) two years later).

	Model 2.Women Adjusted	N_obs_ (N_ind_)	Model 2.Men Adjusted	N_obs_ (N_ind_)
Decision authority		1175 (780)		1312 (840)
DA	1.17 (.74–1.84)		0.56 (.37–.86)**	
PDWT	0.68 (.41–1.12)		0.51 (.30–.87)*	
DA*PDWT	1.28 (.67–2.46)		**1.91 (1.00**–3.63)*	

Social support		1149 (764)		1282 (823)
SS	0.96 (.61–1.50)		1.19 (.79–1.79)	
PDWT	0.53 (.33–.84)**		0.71 (.47–1.07)	
SS*PDWT	**2.22 (1.15**–4.31)*		1.28 (.70–2.33)	

Work-time control				1246 (807)
WTC	1.27 (.80–2.01)	1119 (748)	1.11 (.73–1.68)	
PDWT	0.83 (.55–1.24)		0.73 (.45–1.18)	
WTC*PDWT	0.89 (.45–1.76)		1.15 (.62–2.14)	

Reward		977 (673)		1088 (729)
Reward	0.82 (.50–1.32)		1.23 (.76–1.98)	
PDWT	0.53 (.32–.88)*		0.81 (.47–1.39)	
Reward*PDWT	1.74 (.87–3.52)		0.82 (.41–1.65)	

Note.

Model 2. Adjusted for age, wave, education, marital status, parental status, part-time job, and caring for relative.

PDWT for physically demanding work tasks (physical demands); DA for decision authority; SS for social support; WTC for work-time control.

N_obs_ = number of observations; Nind = number of individuals.

* for *p* < .05; † for 0.05 ≤ *p* < .10.

**Fig. 4:**
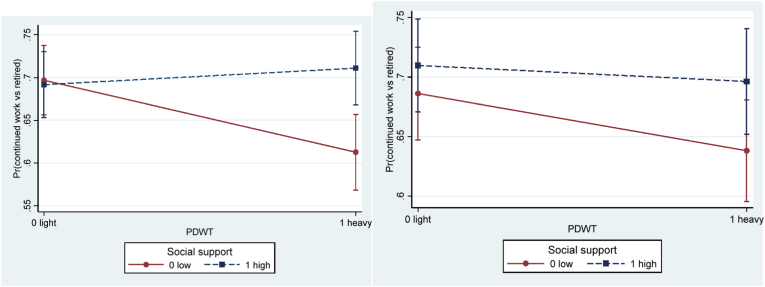
Predictive margins of continued work (vs retired) at follow-up with 95% confidence intervals of interactive effects between physical demands and social support for **a**) women (*p* = 0.018) and **b**) men *(p* = 0.425), in adjusted models. *Note.* PDWT for physically demanding work tasks (physical demands).

Social support borderline significantly moderated the association between physical demands and working longer in the crude model and significantly in the adjusted model (OR = 1.63, 95%CI 1.05–2.53), such that high social support may buffer the negative effect of heavy physical demands on working longer (Fig. 2b). A similar buffering effect of social support was found among women (OR = 2.22, 95%CI 1.15–4.31), but not among men (OR = 1.28, 95%CI 0.70–2.33) (Fig. 4; Table 6).

Neither work-time control nor rewards moderated the association between physical demands and working longer in the crude or the adjusted models (Table 5). Moreover, stratified analyses by gender did not alter any results (Table 6).

### 3.3 Effect of job resources on the association between physical hazards and working longer

No moderating effect of any job resource on the association between physical hazards and working longer was found in the crude or the adjusted models (Table 7). Stratified analyses by gender did not alter any results, except for social support that borderline significantly moderated the association between physical hazards and working longer for men (OR = 1.71, 95%CI 0.92–3.18; *p* = .092; Table 8).

**Table 7:** Interactive effect of job resources and physical hazards in separate models. Odds ratio (OR) and 95% confidence interval (CI) of working longer (continued work (1) vs retired (0) two years later).

	Model 1.Crude	N_obs_ (N_ind_)	Model 2.Adjusted	N_obs_ (N_ind_)
Decision authority		2654 (1687)		2460 (1602)
DA	1.06 (.79–1.42)		1.08 (.79–1.47)	
PHWE	1.04 (.74–1.46)		0.93 (.65–1.34)	
DA* PHWE	0.83 (.54–1.27)		0.78 (.50–1.22)	
Social support		2585 (1655)		2410 (1572)
SS	1.10 (.83–1.47)		1.13 (.84–1.52)	
PHWE	0.84 (.63–1.13)		0.70 (.51–.97)*	
SS* PHWE	1.39 (.91–2.12)		1.44 (.92–2.25)	
Work-time control		2517 (1622)		2347 (1541)
WTC	1.23 (.93–1.63)		1.14 (.84–1.54)	
PHWE	0.94 (.70–1.26)		0.80 (.59–1.10)	
WTC* PHWE	1.09 (.71–1.66)		1.15 (.74–1.79)	
Reward		2190 (1459)		2045 (1386)
Reward	1.07 (.78–1.46)		1.14 (.82–1.58)	
PHWE	0.99 (.70–1.39)		0.89 (.62–1.29)	
Reward* PHWE	0.97 (.61–1.52)		0.90 (.56–1.45)	

Note.

Model 1. Crude (age served as the timing of the event).

Model 2. Adjusted for age, wave, gender, education, marital status, parental status, part-time job, and caring for relative.

PHWE for physically hazardous work environment (physical hazards); DA for decision authority; SS for social support; WTC for work-time control.

* for *p* < .05; † for 0.05 ≤ *p* < .10.

**Table 8:** Interactive effect of job resources and physical hazards in separate models **stratified on gender.** Odds ratio (OR) and 95% confidence interval (CI) of working longer (continued work (1) vs retired (0) two years later).

	Model 2.Women Adjusted	N_obs_ (N_ind_)	Model 2.MenAdjusted	N_obs_ (N_ind_)
Decision authority		1161 (770)		1299 (832)
DA	1.27 (.86–1.89)		0.84 (.50–1.41)	
PHWE	0.65 (.39–1.10)		1.07 (.61–1.85)	
DA#PHWE	1.11 (.55–2.27)		0.75 (.39–1.45)	

Social support		1138 (756)		1271 (816)
SS	1.28 (.87–1.89)		0.95 (.58–1.54)	
PHWE	0.70 (.43–1.13)		0.69 (.44–1.08)	
SS#PHWE	1.18 (.57–2.46)		**1.71 (.92**–**3.18)†**	

Work-time control		1110 (741)		1237 (800)
WTC	1.27 (.85–1.90)		0.99 (.59–1.66)	
PHWE	0.73 (.48–1.13)		0.85 (.51–1.42)	
WTC#PHWE	0.83 (.37–1.83)		1.32 (.68–2.58)	

Reward		965 (662)		1080 (724)
Reward	1.05 (.69–1.61)		1.32 (.72–2.44)	
PHWE	0.70 (.41–1.19)		1.17 (.64–2.14)	
Reward#PHWE	0.93 (.43–2.02)		0.77 (.36–1.63)	

Note.

Model 2. Adjusted for age, wave, education, marital status, parental status, part-time job, and caring for relative.

PHWE for physically hazardous work environment (physical hazards); DA for decision authority; SS for social support; WTC for work-time control.

Nobs = number of observations; N_ind_ = number of individuals. * for *p* < .05; † for 0.05 ≤ *p* < .10.

### 3.4 Age as a moderator on the association between physical demands, job resources, and working longer

**Fig. 5:**
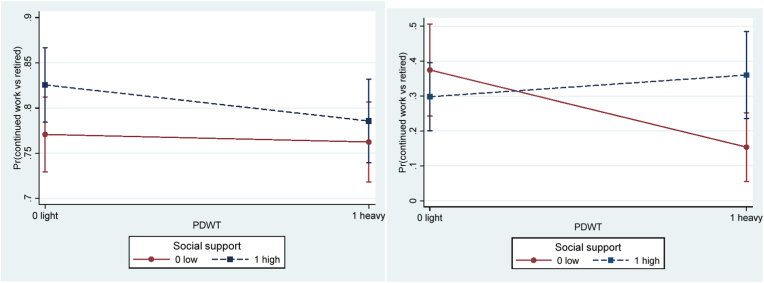
Predictive margins of continued work (vs retired) at follow-up with 95% confidence intervals of interactive effects between physical demands and social support for **a**) 59–63 years old (at baseline) men (*p* = 0.439), and **b**) ≥64 years old men (*p* = 0.018), in adjusted models. *Note.* PDWT for physically demanding work tasks (physical demands).

In Model 3, a three-way interaction term between age category (59–63 versus ≥64 years at baseline), job resource, and physical demands or physical hazards were added to Model 2 (Table 9). A three-way interaction term (OR = 2.78, 95%CI 0.98–7.93) between age category, social support and physical demands on working longer reached borderline significance according to Wald χ^2^−test (3.67, *p* = .055). Stratified analyses by gender showed that the interaction term was only significant for men (OR = 7.02, 95%CI 1.75–28.19; χ^2^ = 7.55, *p* = .006), but not for women (OR = 0.91, 95%CI 0.16–5.16; χ^2^ = 0.01, *p* = .913). Fig. 5 shows the buffering effect of social support on the association between physical demands and working longer for men stratified by age category. Only for the older age category (≥64 years) there was a significant interaction term (OR = 5.07, 95%CI 1.33–19.40; Table 10), such that social support buffered the negative effect of heavy physical demands on working longer. More specifically, Fig. 5b indicates that social support may matter more in cases of heavy compared to light physical demands for working longer among men aged 64 and above. Among women, no such effect was found. However, conclusions about any age effect for women must be taken cautiously, since women that still worked in the older age category (66 years at follow-up) were very few (n = 54; Table 3).

**Table 9:** Wald χ^2^−test for three-ways interactive terms between age category (59–63 versus ≥64 years), job resource, and physical demands (or physical hazards) on working longer (continued work (1) vs retired (0) two years later). Separate models.

Model 3.Job resource	3-way interaction term	Wald χ^2^ (*p*-value) All	Wald χ^2^ (*p*-value) Women^a^	Wald χ^2^ (*p*-value) Men
Decision authority				
	DA* PDWT * age	1.09 (.297)	0.99 (.319)	0.01 (.937)
	DA* PHWE * age	1.84 (.175)	1.48 (.223)	2.46 (.117)

Social support				
	SS * PDWT * age	**3.67 (.055)†**	0.01 (.913)	7.55 (.006)**
	SS * PHWE * age	2.27 (.132)	0.16 (.686)	3.97 (.046)*

Work-time control				
	WTC * PDWT * age	0.15 (.699)	2.69 (.101)	0.99 (.320)
	WTC * PHWE * age	0.00 (.956)	0.21 (.647)	0.83 (.363)

Reward				
	Reward * PDWT * age	0.14 (.708)	**2.83 (.093)†**	1.85 (.174)
	Reward * PHWE * age	**2.77 (.096)†**	1.50 (.221)	0.61 (.436)

Note.

Adjusted for age, gender, wave, marital status, parental status, part-time job, and caring for relative.

PDWT for physically demanding work tasks (physical demands); PHWE for physically hazardous work environment (physical hazards); DA for decision authority; SS for social support; WTC for work-time control.

** for *p* < .01; * for *p* < .05; † for 0.05 ≤ *p* < .10.

a Few observations of women in the age category ≥64 at baseline continued work at the subsequent follow-up wave two years later.

**Table 10:** Stratified analyses on age category (59–63 versus ≥64 years) for men. Interactive effect of social support and physical demands/physical hazards (separate models). Odds ratio (OR) and 95% confidence interval (CI) of working longer (continued work (1) vs retired (0) two years later).

Model 4.	Age category	MenTwo-way interaction term OR (95% CI)	N_obs_ (N_ind_)
PDWT & SS		1 heavy PDWT #1 high SS	
	59–63 years	0.75 (.37–1.55)	1010 (705)
	≥64 years	**5.07 (1.33**–19.40)*	272 (232)

PHWE & SS		1 high PHWE # 1 high SS	
	59–63 years	1.14 (.54–2.42)	1001 (698)
	≥64 years	**4.44 (1.40**–14.12)*	270 (230)

Note.

Adjusted for age, wave, marital status, parental status, part-time job, and caring for relative.

PDWT for physically demanding work tasks (physical demands); PHWE for physically hazardous work environment (physical hazards); SS for social support.

Nobs = number of observations; N_ind_ = number of individuals.

*for *p* < .05.

### 3.5 Age as a moderator on the association between physical hazards, job resources, and working longer

**Fig. 6:**
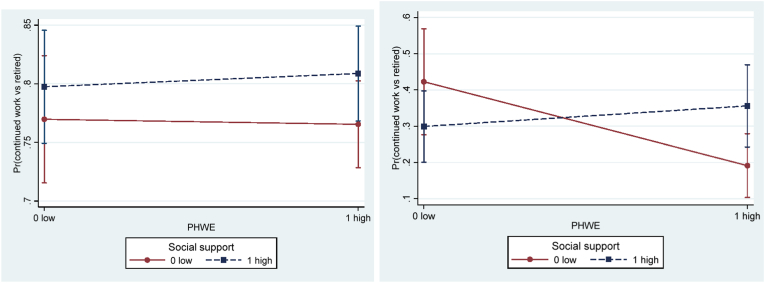
Predictive margins of continued work (vs retired) at follow-up with 95% confidence intervals of interactive effects between physical hazards and social support for **a**) 59–63 years old (at baseline) men (*p* = .732), and **b**) ≥64 years old men (*p* = .012), in adjusted models. *Note.* PHWE for physically hazardous work environment (physical hazards).

A borderline significant three-way interaction term was found between age category, reward and physical hazards on working longer according to Wald χ^2^-test (2.77, p = .096; Table 9). In stratified analyses by gender, the three-way interaction terms did not reach significance, neither among women nor among men. In addition, stratified by gender, a three-way interaction term between age category, social support, and physical hazards on working longer was supported only for men according to Wald χ^2^-test (3.97, p = .046). In the following stratified analyses by age category (Table 10; Fig. 6), a significant interaction term (OR = 4.44, 95%CI 1.40–14.12) was found only for the older age category (≥64 years) of men, implying that high social support buffered the negative effect of high physical hazards on working longer. More specifically, only among individuals with low social support, low physical hazards (vs high) was significantly associated with working longer among men aged 64 years and above (Fig. 6b).

## 4 Discussion

The primary aim of the present study was, in a gender-stratified sample of blue-collar workers, to examine the buffering effects of job resources (decision authority, social support, work-time control, and rewards) on the association of physically demanding work tasks and physically hazardous working environment with working longer. A second aim was to examine whether such relationship was stronger for older workers (64 years and older at the baseline). We found support that decision authority and social support, but not work-time control and rewards, buffered the negative influence of heavy physical demands on working longer. Stratified analyses by gender showed that the buffering effect of decision authority only remained significant for men, while the buffering effect of social support remained significant for women. With regard to age effects on the studied associations, the buffering effects of social support on the association of both heavy physical demands and high physical hazards with working longer were found only for men who had turned at least 64 years at baseline.

### 4.1 Associations between physically demanding work tasks and working longer

Unlike previous research (Andersen et al., 2021), our results suggest that decision authority may buffer the influence of heavy physical demands on working longer, such that, only in cases of low decision authority, there was a negative influence of heavy compared to light physical demands on working longer. However, the previous study included both blue- and white-collar workers, and the comparisons were made between seated and physically active job. As a consequence, their comparisons to a large extent concern differences between white- and blue-collar workers. By solely targeting blue-collar workers allowed us to examine the buffering effects of job resources on heavier (the upper half) versus lighter (the lower half) physical demands. Our finding suggests that proper decision authority may enable workers to work longer even if they have heavy physical demands. For example, it can be possible to perform physically demanding tasks in a more comfortable and more adapted way to one's physical conditions. In a similar vein, one recent cross-sectional study including mostly blue-collar workers above 50 years of age, who all had heavy physical demands, found that low job resources were associated with higher perceived stress (Vinstrup et al., 2021). However, stratifying by gender, we found that the buffering effect of decision authority only remained significant for men. Supplementary analyses showed that 1) among men who had low decision authority, those with light compared to heavy physical demands had higher probability of continuing working and 2) among men who had light physical demands, those with low compared to high decision authority had higher probability of continuing working. The former finding was expected, whereas the latter finding was a bit surprising. Perhaps, among men with light physical demands, those with low decision authority are often at a low job position and with low income as compared with those with high decision authority, and they might not afford to retire. At the same time, they still can manage their work as the physical demanding is low, which give them the possibility to stay in the labour market longer.

Also, we found support that social support buffered the influence of physical demands on working longer, such that high social support enabled the workers to work longer disregarded of having heavy or light physical demands. For workers with heavy physical demands, social support seems to promote working longer, which suggests that social support from managers and colleagues really might buffer against the negative consequences from heavy physical demands. Literature largely supports that social support at work from co-workers and managers have beneficial effects on wellbeing and health (Kay-Eccles, 2012; Velando-Soriano et al., 2020). Stratified analyses by gender, this buffering effect of social support was only found among women.

In line with Andersen et al. (2021), we did not find any buffering effect of rewards (from management) on the association between physical demands and working longer. Neither did we found any support that work-time control buffered the influence of physical demands on working longer. This suggests that neither rewards nor work-time control may be sufficient to compensate for heavy physical demands on the decision to remain in or leave the work force. However, other studies show that especially older workers benefit from high work-time control in terms of health (Albrecht et al., 2020) and the possibility to work longer (Platts et al., 2021; Stengård et al., 2021). Future research should further scrutinize the benefits of work-time control for longer working lives.

### 4.2 Associations between physically hazardous working environment and working longer

With regard to physically hazardous working environment, none of the examined resources were found to buffer the influence of physical hazards on working longer. This suggests that none of the examined resources can overall compensate for high physical hazards, such as, noise, excessive heat or cold, and other hazardous condition with risk for injuries, on the decision to remain in the work force. To be noted, we do not know whether an individual stayed or changed job position between baseline and follow-up, and thus, whether a potential job change improved the working conditions.

### 4.3 Age as a moderator

Finally, with regard to interaction effect of age, a buffering effect of social support was found on the association between physical demands and working longer, such that, high social support enabled *men* who had reached the age of 64 to work longer regardless of whether they had heavy or light physical demands. To be noted, for women, such buffering effect of social support was supported regardless of age. This suggests that for working longer—for women and older men—social support at work is of importance for buffering heavy physical demands. Thus, factors like pleasant atmosphere and spirit of unity at the workplace, where colleagues are there for each other and cohesiveness among co-workers and managers, may compensate or counteract heavy physical demands and contribute to remaining longer in the work force. With regard to physical hazards, a similar buffering effect was found for men who had reached the age of 64, suggesting that high social support may allow older men to work longer regardless of having high or low physical hazards. For comparisons, another study also found that social support could buffer the effect of operational demands among policemen on depression (Baka, 2020).

In our analyses, we did not control for health or work abilities since these may mediate the associations between physical demands/physical hazards or job resources and retirement timing, and hence may over adjust the examined relationships. However, in sensitivity analyses, we included self-rated health and physical and psychological work abilities as covariates in the analyses (results not reported). For social support, all reported associations still were significant, although the buffering effect of decision authority on the association between physical demands and working longer, did not reach significance level maybe due to over adjustment or reduced power. This strengthens our conclusion that social support at work is an important job resource, which buffers the influence of heavy physical demands on retirement timing.

### 4.4 Strength and limitations

A strength of this study is that we for hypothesis testing utilized a large approximately representative longitudinal cohort study of the Swedish working population. Moreover, that we targeted blue-collar workers, which often face physically demanding working conditions, and moreover stratified the analyses by gender. Also, we used several well-established and well-tested measures for job resources. Nevertheless, the reliability of the seven-item scale measuring rewards could be questioned, as Cronbach's alpha was a bit low, 0.64 compared to the commonly acceptable value of 0.70. Possibly some of the items about job security and promotion prospects may be less valid for the oldest workers. This means that the rewards measured may not be that meaningful to the population in question and any true associations with rewards may be harder to discover. As the reward scale is a well-established and frequently used measure (Li et al., 2019), we decided against modifying it, but we suggest that future research should evaluate, validate and develop the scale for the population of older workers.

To be noted, the median split of decision authority resulted in a rather uneven split: with the observations with the 62.5% highest versus the 37.5% lowest values. This differs from the other examined job resources, where the division by median value resulted in approximately two equal-sized categories representing values of high and low job resource.

Also, because in the present study a particular baseline and its follow-up wave are two years apart, we do not know the exact date and age of retirement for a person that made a transition from work to retirement, and therefore the two age categories are not mutually exclusive. For instance, a person (observation) in the younger age group and a person (observation) in the older age group that had retired at follow-up, may in fact for some instances retired at the exact same age. This implies that more research is warranted on any age differences of the importance of buffering job resources on the association between physical demands (and physical hazards) and retirement timing, where more specific retirement dates could be utilized.

Finally, logistic regression demands a lot of statistical power for detecting associations, but there are no agreed standardized recommendations/rules on how to calculate a sufficient sample size as it depends on many factors. However, according to recommendation brought forward of both Harrell (2015) and Bujang et al. (2018), even our most complex analysis (the gender-stratified three-ways interaction analysis) reach sufficient power (Bujang et al., 2018; Harrell, 2015). However, one must bear in mind that among women, conclusions about any age effect must be taken cautiously, since very few women in the older age category still worked at follow-up. Consequently, while it would have been good to adjust the statistical significance level with for example, Benjamini-Hochberg adjustments, we decided against it despite the risk for false positives. A reason for that is the rather limited sample size; also, we deem the risk of creating false negatives with such adjustments as more concerning. In addition, our findings show a consistent pattern, where social support appears to be the important resource to counteract high physical demands (which is in line with earlier research on psychosocial work conditions and disability pension (Leineweber et al., 2019)).

## 5 Conclusions

The present study found that social support and to some extent decision authority may buffer the influence of physically demanding work tasks on retirement timing for blue-collar workers, such that, proper social support or control over one's work tasks may enable individuals to work longer regardless of the degree of physical demands. The buffering effect of social support seem to be particular pertinent for women in all ages (from 59 years) and for older men (from 64 years). These findings suggest that for women employees, who in Sweden predominantly work in the public health and social care sectors, social support from managers, teams and colleagues, is a crucial determinant for continuing working, especially when facing high physical demands. But for men (who often work in the industry and transport sector or as craftsmen) social support seems to be important to counteract both high physical demands and physical hazards only when approaching the Swedish normative retirement age of 65 years. In addition, for men, high levels of decision authority at their work may increase their likelihood to continue working even with high physical demands jobs. Thus, to prolong working life among blue-collar workers with heavy physical demands (and to some extent with high physical hazards), it seems important for organisations to invest in a good social work environment with high social support from both managers, teams and colleagues. More research is warranted to study whether other resources could buffer physically demanding or hazardous work situations and to confirm the age difference between men and women regarding the influence of social support on working longer.

## Ethical statement

This study has been approved by the Regional Research Ethics Board in Stockholm, document numbers: 2020/01968. Participants of the Swedish Longitudinal Occupational Survey of Health (SLOSH) received written information on the survey, and return of the survey indicated informed consent.

## Author statement

**J Stengård**, **C Leineweber** and **H-X Wang** planned and conceptualized the study. JS performed the statistical analyses, interpreted the results, and drafted the manuscript. All authors contributed to interpreting the results, revising the manuscript and approved the final version of the manuscript.

## Funding

This work was supported by the 10.13039/501100006636Swedish Research Council for Health, Working Life and Welfare (FORTE) under Grant 2019–01120. Data collection was supported by the 10.13039/501100006636Swedish Research Council for Health, Working Life and Welfare (FORTE) through the 10.13039/501100013346Stockholm Stress Center under Grant 2009–1758; the 10.13039/501100006636Swedish Research Council for Health, Working Life and Welfare under Grant 2005–0734; and the 10.13039/501100004359Swedish Research Council (VR) under Grant 2009-06192, 2013–01645, 2015–06013, and 2017–00624. The funding sources had no role in the writing of the manuscript or the decision to submit it for publication.

## Declaration of competing interest

None.

## Data Availability

The authors do not have permission to share data.
